# Paricalcitol and hydroxychloroquine modulates extracellular matrix and enhance chemotherapy efficacy in pancreatic cancer

**DOI:** 10.1038/s41417-025-00967-9

**Published:** 2025-09-27

**Authors:** Dhana Sekhar Reddy Bandi, Sujith Sarvesh, Jeremy Foote, Doug Welsch, Changde Cheng, Mehmet Akce, Ganji Purnachandra Nagaraju, Bassel F. El-Rayes

**Affiliations:** 1 Department of Hematology and Oncology, University of Alabama at Birmingham, Birmingham, AL USA; 2 Department of Microbiology, University of Alabama at Birmingham, Birmingham, AL USA; 3 O’Neal Comprehensive Cancer Center, Heersink School of Medicine, University of Alabama at Birmingham, Birmingham, AL USA; 4 Institute for Cancer Outcomes and Survivorship, Heersink School of Medicine, University of Alabama at Birmingham, Birmingham, AL USA; 5 Department of Biomedical Informatics and Data Science, Heersink School of Medicine, University of Alabama at Birmingham, Birmingham, AL USA

**Keywords:** Pancreatic cancer, Targeted gene repair

## Abstract

Pancreatic ductal adenocarcinoma (PDAC) is a highly aggressive cancer with poor prognosis and limited therapeutic options. In a previous publication, our group defined some of the mechanisms that vitamin D analogue paricalcitol (P) and hydroxychloroquine (H) potentiated the effects of gemcitabine-based chemotherapy in PDAC. Based on this, we hypothesized that PH may potentiate 5-fluorouracil (5FU) and Oxaliplatin-based chemotherapy, and this may involve a novel mechanism of extracellular matrix (ECM) modulation. The combination of PH with 5FU+Oxaliplatin significantly increased the cell death, apoptosis, and S-phase cell cycle arrest as compared to untreated or 5FU + Oxaliplatin-treated MIA PaCa-2, HPAC and KPC cell lines. In vivo, the combination therapy inhibited PDAC growth and altered the immune landscape by activating T and NK cells. Proteomic analysis revealed significant reduction in ECM proteins, specifically integrin beta-4 (ITGB4). Confirmation of the role of ITGB4 was performed through genetic knockdown of ITGB4, which led ECM inhibition. In conclusion, the combination of PH significantly enhances the efficacy of Oxaliplatin and 5FU. We identified a new mechanism of action of PH through inhibiting ITGB4, leading to ECM modulation. These results suggest that the combination of PH with cytotoxic chemotherapy should be tested in PDAC clinical trials.

## Introduction

Pancreatic ductal adenocarcinoma (PDAC) is one of the most deadly malignancies [1]. It is estimated that by 2030, PDAC will become second leading cause of cancer-related deaths in the U.S. The 5-year survival rate for PDAC is poor (~11%) due to its tendency for early metastasis and resistance to systemic treatments, including cytotoxic and immune-based therapies [2, 3]. The resistance to systemic therapies is partly due to the presence of collagen-rich dense extracellular matrix (ECM) [4].

The PDAC ECM consists of several components, such as collagens, glycoproteins, fibronectins, proteoglycans, hyaluronic acid, and laminins. Cancer-associated fibroblasts (CAF) play a pivotal role in the development and maintenance of the ECM. PDAC cells interact with ECM components through integrin’s and receptor tyrosine kinases to form focal adhesion complexes, which help in growth, metastasis, and drug resistance [5, 6]. ECM affects the behavior of immune and angiogenic cells in the tumor microenvironment (TME), promoting a tumorigenic effects [7]. The compact and heterogeneous physical network of ECM components inhibit drug delivery, further contributing to resistance and poor prognosis [8]. Therefore, the ECM has pleotropic effects in PDAC, facilitating cancer cell survival, metastasis, and chemo-resistance. Modulating the ECM represents a rational approach to inhibit PDAC progression and promote the effects of systemic therapies.

Previous reports have indicated that vitamin D analogs, such as paricalcitol (P) and hydroxychloroquine (H) have effects on CAF and immune TME. Vitamin D analogs has been shown to suppress activated CAF in PDAC and acts as master transcriptional regulator of pancreatic stellate cells (PSCs) to reprise in the quiescent state resulting in stromal remodeling [9, 10]. It has also been shown to suppress the expression of several ECM proteins, such as smooth muscle alpha-actin (α-SMA) in breast cancer [11–14]. H selectively reduced the deposition of ECM in hepatic stellate cells by inhibiting α-SMA and collagen I [15]. H alleviates renal interstitial fibrosis by inhibiting PI3K/Akt signaling and attenuates ECM and EMT by suppressing NF-κB signaling [16]. H has also been shown in clinical trials to modulate the immune microenvironment in PDAC [17]. The effects of the combination of H and P with 5FU-based chemotherapy on modulation of ECM and immune microenvironment in PDAC has not been previously studied.

Clinical trial targeting ECM components, including matrix metalloproteinase or hyaluronidase in PDAC have resulted in limited success [18]. The heterogeneity of CAF’s and redundant ECM composition and structure limits the benefit of these therapies, which are very specifically designed against one component or pathway in the ECM. PDAC cells frequently develop resistance mechanisms, such as alterations in integrin expression and ECM remodeling, which reduce the effectiveness of such treatments. In our prior study, we reported the addition of HP to gemcitabine resulted in immune modulation and effects on autophagy [19]. These effects were observed in preclinical models and clinical samples from paired biopsies obtained from patients on a trial [19]. While gemcitabine-based therapy is a standard chemotherapy for PDAC, accumulating evidences suggests that 5-FU-based regimens may have shown superior efficacy [20]. Encouraged by the observed effects of PH with gemcitabine, we planned to assess this combination with 5FU-based therapy. In addition, our objective was to evaluate if the mechanisms involved in potentiation of 5FU-based therapy by H and P were mediated through mechanisms involving modulation of immune cells and ECM proteins in the TME.

## Results

### Paricalcitol and hydroxychloroquine enhances the growth inhibitory effects of 5FU and Oxaliplatin in both murine and human PDAC cells

**Fig. 1:**
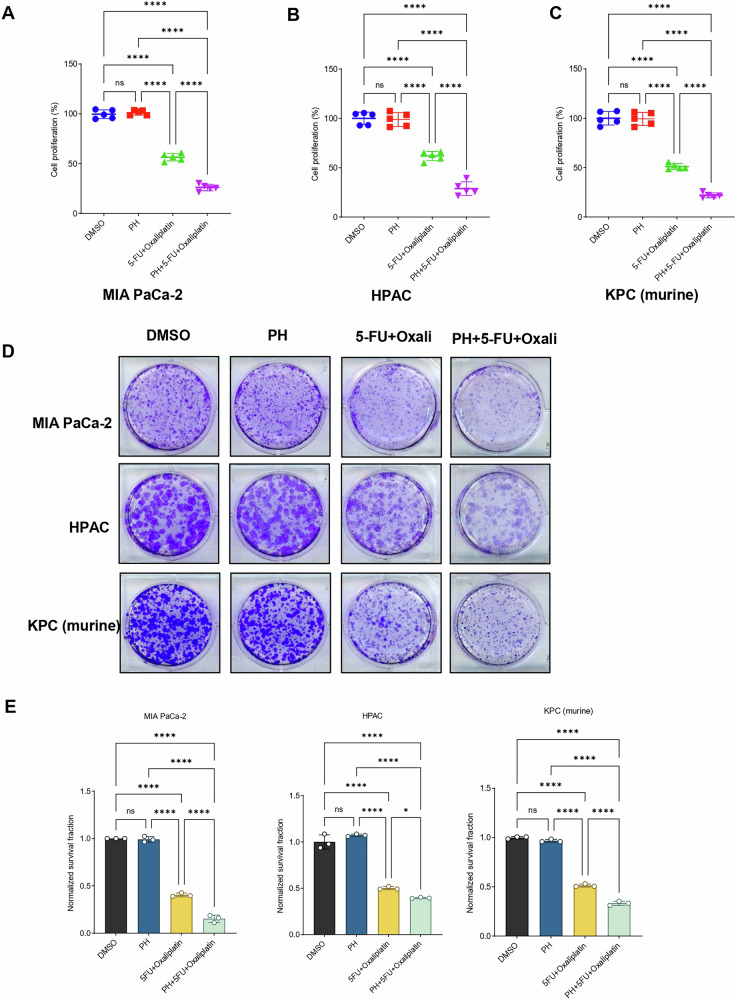
PH potentiates the growth inhibition of 5FU and Oxaliplatin in PDAC cells. **A**–**C** The indicated cancer cell lines were treated with various concentration of 5FU (15 μM) and Oxaliplatin (25 μM), paricalcitol (350 nM), and hydroxychloroquine (25 μM) for 3 days and subjected to MTT assays. Relative percentage cell viability was plotted with respect to DMSO treated cells. **D** The indicated cancer cell lines were treated with DMSO, PH, 5FU+oxali, and combination (PH + 5FU+Oxali) for 2–4 weeks, and long-term cell survival was measured using clonogenic assays. Representative images are shown. **E** Colony counts from the clonogenic assay data shown in (**D**). Data represent the mean ± standard error of three biological replicates. Statistical significance was assessed using two-way ANOVA. ns not significant, *p < 0.05, ****p < 0.0001.

The effects of PH in combination with 5FU and Oxaliplatin (oxali) on murine and human PDAC cells were evaluated by dosing the cells with varying concentrations of the drugs and then quantifying the survival rates by cell viability using MTT assay. Human PDAC cell lines MIA PaCa-2 and HPAC, and murine KPC PDAC cells were cultured in the presence of PH, 5FU+Oxali either alone or in combination for 72 h (Fig. 1A–C). The combination treatment was extremely efficient in urging cytotoxicity in all the cell lines. The combination index (CI) values, calculated with PRISM software for dose-effect analysis, designated the presence of a robust synergism between 5FU + Oxali with PH, exclusively at lower and intermediary drug doses compared to chemotherapy alone. We then asked whether this potential combination has effect on long-term survival of PDAC cells using clonogenic assays. Treatment of PH with 5FU (10 µM) and Oxali (20 µM) inhibited colony-forming ability of PDAC cells (Fig. 1D, E). In addition to this, we have also checked if PH sensitizes the combination of 5FU, Oxaliplatin, and Irinotecan in PDAC. To this end, we have performed MTT assay in MIA PaCa-2, HPAC, and murine KPC cells and found that the combination treatment significantly suppressed cell growth compared to chemotherapy alone (Supplementary Fig. 1A–C). This increased suppression suggests that PH may acts as chemosensitzer. Furthermore, clonogenic assay revealed that the combination of 5FU, Oxaliplatin, and Irinotecan resulted in marked reduction in colony formation compared to any other treatment group, indicating substantial impairment in the long-term proliferative capacity (Supplementary Fig. 1D, E).

### PH with 5FU and Oxaliplatin combination promotes apoptosis in PDAC cells

**Fig. 2:**
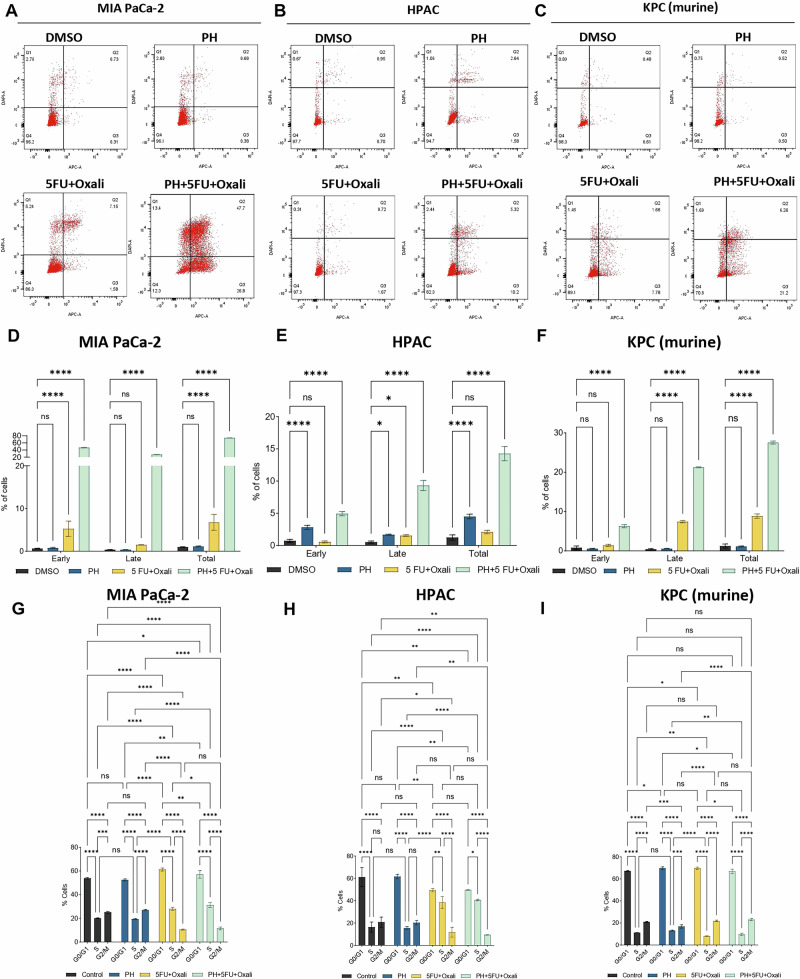
PH potentiates 5FU and Oxaliplatin treatment induces apoptosis and S-phase arrest in PDAC cells. **A**–**C** The indicated PDAC cell lines were treated with DMSO, PH, 5FU + Oxali, and PH + 5FU + Oxali for 72 h, and apoptosis was measured using an annexin V/propidium iodide staining kit. **D**–**F** The early, late, and total apoptotic population from the shown data in (**A**–**C**) is plotted in DMSO, PH, 5FU + Oxali, and combination (PH + 5FU + Oxali) treated cells. **G**–**I** The cell cycle distribution of MIA PaCa-2, HPAC, and KPC cells treated with DMSO, PH, 5FU + Oxali, and PH + 5FU+Oxali analyzed by flow cytometry. Data represent the mean ± standard error of three biological replicates. Statistical significance was assessed using two-way ANOVA. ns not significant, *p < 0.05, ^∗∗^*p* < 0.01, ^∗∗∗^*p* < 0.001, and ****p < 0.0001.

We analyzed the effect of this combination on inducing apoptosis in PDAC cells. Apoptosis measurement by annexin V and PI staining method. In MIA PaCa-2 cells, apoptosis was significantly higher in the combination arm (PH + 5FU + Oxali) compared to DMSO, PH, and 5FU + Oxali alone (Fig. 2A, D). Similar results were seen in HPAC (Fig. 2B, E) and KPC cells (Fig. 2C, F). Taken together these results prove that the combination treatment is significantly enhancing apoptotic effect in both human and murine PDAC cells highlighting potentiation of 5FU+Oxali by PH in PDAC treatment comparing to existing treatment.

### PH with 5FU and Oxaliplatin combination inhibits cell cycle arrest in PDAC cells

To study the anti‑proliferative mechanism of 5FU + Oxali combined with PH, the effect of this combination treatment on the PDAC cell cycle was studied. Briefly, KPC, MIA PaCa-2, and HPAC cells were treated with 5FU (10 µM) and Oxaliplatin (20 µM), 0.35 µM of P and 25 µM of H alone or in combination for 72 h (Fig. 2G–I and Supplementary Fig. 2A–C). 5FU+Oxali alone or in combination with PH resulted in S phase arrest. This is an effect that did not occur in DMSO or PH treated cells (Fig. 2G–I and Supplementary Fig. 2A–C). The S phase arrest is due to effects of 5FU + Oxali and is similar to effects previously reported [21, 22].

### PH with 5FU and Oxaliplatin combination more potently inhibits PDAC tumor growth

**Fig. 3:**
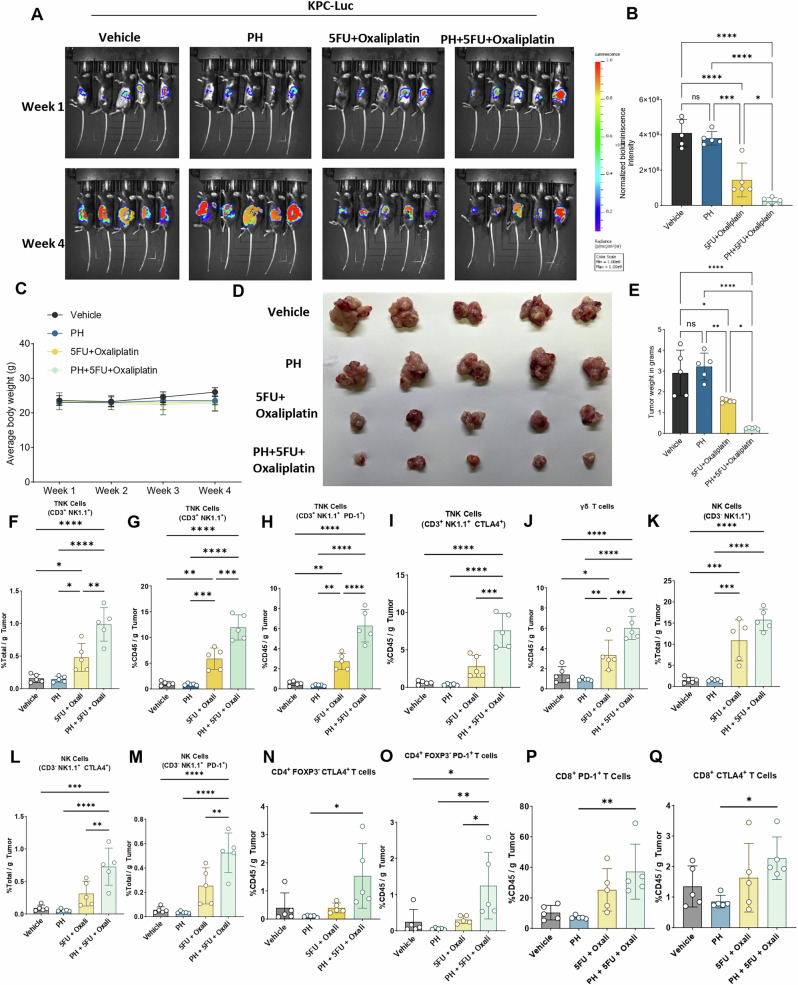
The addition of PH to 5FU and Oxaliplatin combination treatment significantly inhibits PDAC growth in an orthotopic pancreatic implantation model and activates immune-mediated responses. **A** *F*-luc-labeled KPC cells were orthotopically implanted into C57BL/6J mice (n = 20). After 1 week, group of 5 mice were orally administered vehicle, PH, 5FU + Oxali or PH + 5FU + Oxali. Bioluminescence images at the indicated time points are shown. **B** Normalized quantification of bioluminescence intensities for (**A**). **C** Average body weights of the animals during the treatment shown in experiment (**A**). **D** Tumor images at the end of the experiment are shown. **E** Tumor weights for the image shown in (**D**). **F** % of TNK cells (CD3^+^ NK1.1^+^) in total tumor tissues (n = 5). **G** % of TNK cells (CD3^+^ NK1.1^+^) in per gm of CD45^+^ cells (n = 5). **H** % of PD-1^+^ cells in CD3^+^ NK1.1^+^ TNK cells in per gm of CD45^+^ cells (n = 5). **I** % of CTLA4^+^ cells in CD3^+^ NK1.1^+^ TNK cells in per gm of CD45^+^ cells (n = 5). **J** % of γδ T cells per gm of CD45^+^ cells. **K** % of NK cells per gm of total tissue. **L** % of CTLA4^+^ cells in NK cells in per gm of CD45^+^ cells (n = 5). **M** % of PD-1^+^ cells in NK cells in per gm of CD45^+^ cells (n = 5). **N** % of CTLA4^+^ cells in CD4^+^ T cells (n = 5). **O** % of PD-1^+^ cells in CD4^+^ T cells (n = 5). **P** % of PD-1^+^ cells in CD8^+^ T cells (n = 5). **Q** % of CTLA4^+^ cells in CD8^+^ T cells (n = 5). Statistical significance was assessed using two-way ANOVA. ns non-significant, ^∗^*p* < 0.05, ^∗∗^*p* < 0.01, ^∗∗∗^*p* < 0.001, and ^∗∗∗∗^*p* < 0.0001.

Since PH has effects on TME as well as cancer cells, orthotopic mouse model of PDAC were used to further evaluate this regimen. Mice were treated with vehicle, PH, 5FU + Oxali or combination. The combination therapy significantly inhibited PDAC growth (Fig. 3A, B) compared to vehicle. Additionally, the combination therapy demonstrated significantly greater efficacy compared to either 5FU + Oxali or PH alone. The body weight of all mouse groups was stable; suggesting all treatment were well tolerated (Fig. 3C). End-of-study KPC-luc tumor pictures and weight confirmed the potentiation of 5FU+Oxali by PH (Fig. 3D, E). Variance between the four groups (n = 5) was evaluated and found to be comparable.

### The combination of PH with 5FU and Oxaliplatin alters immune landscape in the KPC-luc-TME

To assess the immune effects of this regimen, we assessed the quantities of T and NK cell subsets by multiparameter flow cytometry. The combination of PH with 5FU + Oxali significantly activated T and NK cells population in total tumor well as in only CD45^+^ cells compared to vehicle, 5FU + Oxali and PH. In addition, we noticed a significant increase in the proportions of CD3^+^ NK1.1^−^ PD-1^+^, and CD3^+^ NK1.1^−^ CTLA-4^+^ TNK cells, suggesting that the combination therapy not only enhances the TNK cells activation but also promotes an immune environment favorable to antitumor responses in the KPC-luc TME (Fig. 3F–I). A substantial increase in the proportions of γδ T cells in the combination treatment compared to any other treatments was observed (Fig. 3J). Significant activation of NK cells in the combination treatment as compared to vehicle, PH and 5FU + Oxali was observed (Fig. 3K). Increased expression of CD3^−^ NK1.1^+^ CTLA-4^+^ and CD3^−^ NK1.1^+^ PD-1^+^ NK cells in the combination treatment suggests a potential shift in the immune profile (Fig. 3L, M). Combination of PH with 5FU+Oxali did not have any significant impact on the CD4^+^ and CD8^+^ T cells (Supplementary Fig. 3A–F). An increase in the expression of CD4^+^ FOXP3^−^ CTLA-4^+^ (Fig. 3N) and CD4^+^ FOXP3^−^ PD-1^+^ (Fig. 3O) (non-significant) in combination treatment was observed, suggesting the combination resulted in exhaustion and increase in regulatory capacity of CD4^+^ T cells. No change in the expression of CD8^+^ FOXP3^−^ PD-1^+^ (Fig. 3P) and CD8^+^ FOXP3^−^ CTLA-4^+^ (Fig. 3Q) was observed in combination treatment.

### The combination of PH with 5FU and Oxaliplatin inhibits ECM formation through ITGB4

**Fig. 4:**
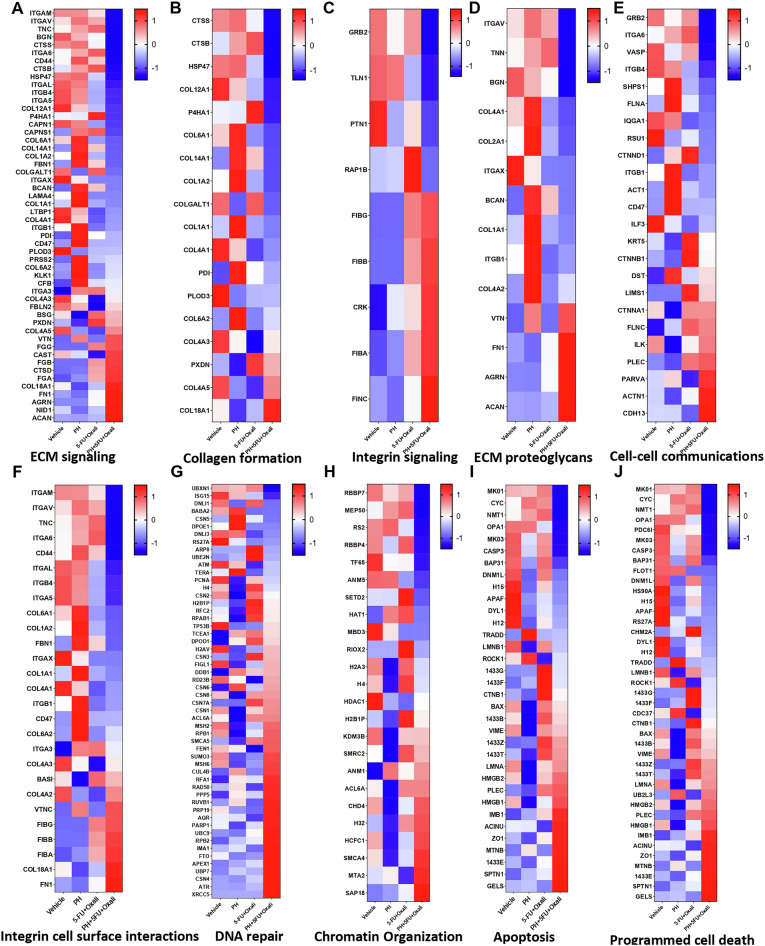
The addition of PH to 5FU and Oxaliplatin alters protein expression profiles in PDAC, influencing key pathways including ECM signaling, DNA repair, and apoptosis. **A** cluster heat map of proteomic profiles through four experimental conditions is shown, with each row representing a different protein and each column corresponding to a sample group. The heat map displays differential protein expression levels, where regions in red indicate increased expression and blue regions indicate decreased expression. White areas indicate no change in expression. The conditions shown are DMSO, PH, 5FU + Oxali, and PH + 5FU + Oxali. **A** Cluster heat map of ECM signaling profile. **B** Cluster heat map of Collagen formation profile. **C** Cluster heat map of ECM proteoglycans profile. **D** Cluster heat map of integrin signaling profile. **E** Cluster heat map of cell-cell communications profile. **F** Cluster heat map of integrin cell surface interactions profile. **G** Cluster heat map of DNA repair profile. **H** Cluster heat map of chromatin organization profile. **I** Cluster heat map of apoptosis profile. **J** Cluster heat map of programmed cell death profile.

As the tumor ECM plays major role in PDAC progression, we then asked if the combination treatment has impact on ECM inhibition. To this end, we utilized mass spectrometry (MS)-based proteomics on mouse models of PDAC treated with DMSO, PH, 5FU + Oxali, and combination. Since the matriosome includes several of ECM protein such as collagens, matrix metalloproteinase (MMP), glycoproteins, and proteoglycans, we were interested to uncover the changes on these proteins in combination treatments [5]. In our study, PH with 5FU+Oxali treatment caused a decreased abundance of several ECM-related proteins. Inhibition in collagen formation proteins such as COL1A1, COL2A1, COL4A1, COL161, COL9A1, COL12A1, COL14A1, CTSB, HSP47, TNC, and BCAN was observed in the animals treated with the combination (Fig. 4A). This suggests changes in the tumor progression and alterations in the ECM remodeling and tumor stroma interactions. We further extended our analysis to check if this inhibition was mediated through integrin signaling and identified that several integrin signaling proteins, along with collagen proteins, were significantly inhibited in the combination treatment, including ITGB4, ITGAL, ITGAM, ITGA5, and ITGAV (Fig. 4B, C). This result points to a potential mechanism by which the combination treatment disrupts integrin-mediated pathways that are necessary for the ECM stability and tumor progression in PDAC. Examining the ECM proteoglycan signaling identified several collagen-binding proteins such as COL4A1, COL2A1, COL1A1 along with integrin’s (ITGB and ITGAV) were reduced in combination treatment (Fig. 4D). Results of cell-cell communication revealed reduction of GRB2, ITGB4, SHPS1, FLNA, RSU1, and CD47, which impairs cell proliferation, adhesion, migration, and immune evasion. Activation of CDH13, PARVA, ILK, and FLNC enhances cell adhesion, migration, and survival, potentially driving tissue remodeling (Fig. 4E).

Proteins such as ITGAM, ITGAV, ITGA6, ITGB4, ITGB1, ITGA5, and ITGA3, along with ECM-related genes like TNC, COL1A1, COL1A2, COL6A1, COL6A2, FBN1, COL4A1, ITGAX, CD47, and CD44, play critical roles in regulating cell-matrix interactions and ECM remodeling. The downregulation of the expression of these genes in the combination treatment arm likely impairs key cellular processes, including adhesion, migration, and signaling, potentially affecting tissue structure and cellular responses to the treatment (Fig. 4F). In combination treatment, the downregulation of the expression of various genes involved in DNA repair and signaling, including UBXN1, ISG15, DNLI1, BABA2, CSN5, DPOE1, DNLI3, RS27A, ARP8, UBE2N, ATM, TERA, PCNA, H4, and CSN2, points to a potential impairment in the DNA damage response and repair mechanisms (Fig. 4G). These proteins are critical for various aspects of DNA repair, including ubiquitination, DNA damage sensing, repair pathway activation, and cell cycle regulation. In addition, DNA repair pathways are key mechanisms of resistance to DAN damaging agents such as Oxaliplatin. Combination treatment potently inhibited the expression of chromatin organization proteins such as RBBP7, MEP60, RBBP4, SETD2, and HAT1 (Fig. 4H). Reduced expression of apoptosis proteins like CASP3, Cytochrome C, TRADD, MAPK1, MAPK3, and APAF1, and activation of pro-apoptotic protein such as BAX were observed in the combination treatment confirming the apoptosis induction observed in the PDAC cell line experiments (Fig. 4I). Programmed cell death proteins such as CYC, NMT1, OPA1, CASP3, and APAF1 found to be downregulated in combination treatment (Fig. 4J).

### Pharmacological and genetic suppression of ITGB4 inhibits PDAC growth and ECM formation by COL1A1

**Fig. 5:**
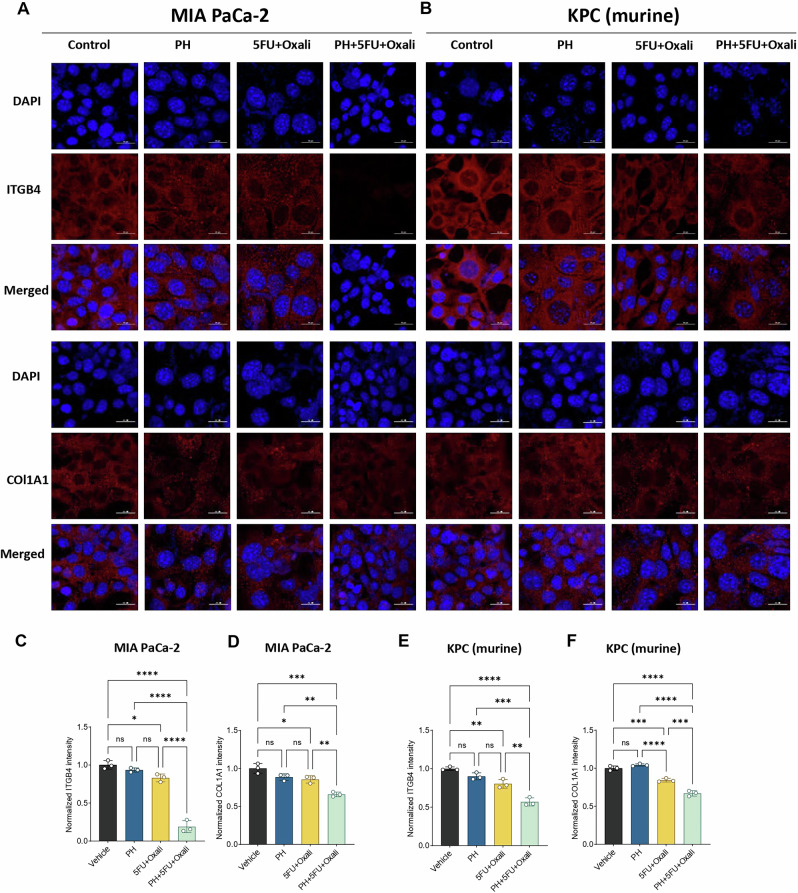
Immunofluorescence analysis of effects of adding PH to 5FU Oxaliplatin on ITGB4 and COL1A1 expression in PDAC cells. **A** Immunofluorescence showing the expression of ITGB4 (red) in human MIA PaCa-2 cells treated with DMSO, PH, 5FU + Oxali, or PH + 5FU + Oxali for 72 h. Nuclei are stained with DAPI (blue), and the merged image shows the overlap of ITGB4 expression and DAPI staining. **B** Immunofluorescence showing the expression of ITGB4 (red) in murine KPC cells treated with DMSO, PH, 5FU + Oxali, or PH + 5FU + Oxali for 72 h. Nuclei are stained with DAPI (blue), and the merged image shows the combined localization of ITGB4 and DAPI. Quantification of ITGB4 and COL1A1 fluorescence intensity in MIA PaCa-2 (**C**, **D**) and KPC (**E**, **F**) and cells treated with DMSO, PH, 5FU + Oxali, or PH + 5FU + Oxali for 72 h. The intensity was normalized to DAPI staining for each sample. Data are presented as mean ± SD. Statistical significance was assessed using two-way ANOVA. ns non-significant, *p < 0.05, **p < 0.01, ***p < 0.001, ****p < 0.0001.

**Fig. 6:**
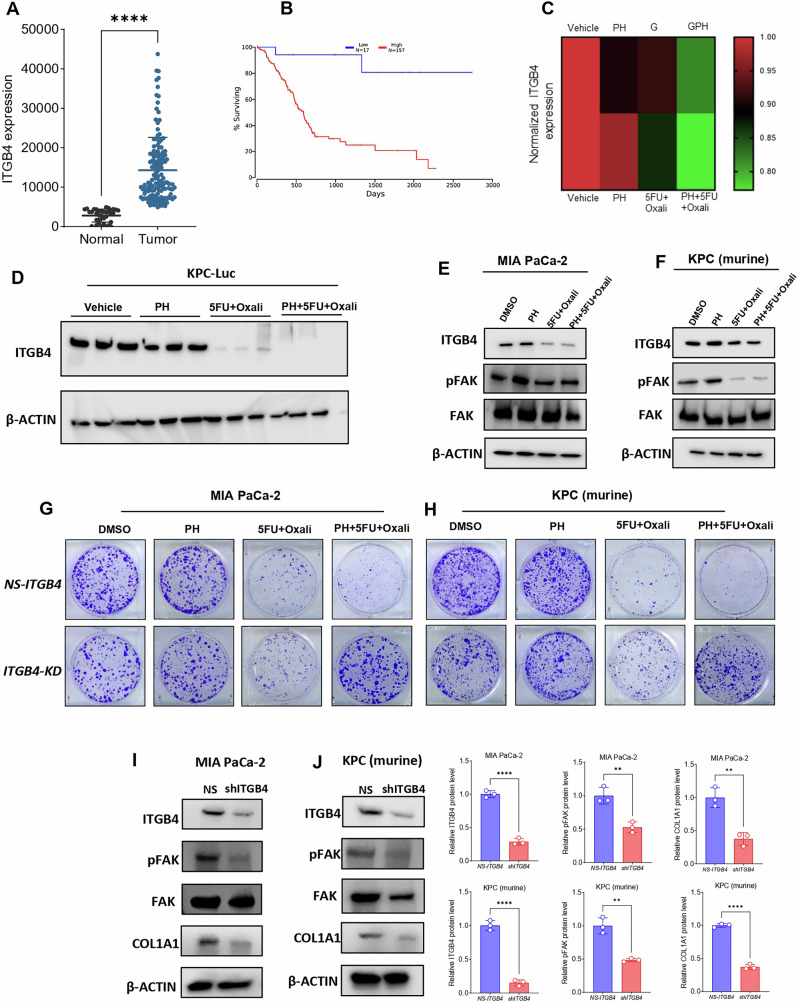
Impact of ITGB4 colony formation, sensitivity to combination therapy, and expression of pFAK and COL1A1 in human and murine PDAC cells and on outcome of patients with PDAC. **A** Expression levels of ITGB4 in normal and PDAC tissues in TCGA. **B** Kaplan plot showing overexpression of ITGB4 associates with poor prognosis in terms of overall survival in PDAC patients. **C** Heat map depicting the expression comparisons of ITGB4 in gemcitabine and 5FU + oxali treatments in combination with PH. **D** Western blot results showing the complete inhibition of ITGB4 in tumor tissues from the KPC orthotopic experiment shown in Fig. 3. **E** Immunoblotting showing the reduced expression levels of ITGB4, phospho FAK, and unchanged levels of total FAK in MIA Paca-2 cells treated with DMSO, PH, 5FU + oxali and PH + 5FU + oxali treatments for 72 h. **F** Immunoblotting showing the reduced expression levels of ITGB4, phospho FAK, and unchanged levels of total FAK in murine KPC cells under the same conditions as in (**E**). **G** Human PDAC cells with ITGB4 KD (MIA PaCa-2) exhibit reduced colony formation and are less sensitive to combination treatment with PH + 5FU + Oxali compared to single treatments (DMSO, PH, and 5FU + Oxali). **H** Murine PDAC cells (KPC) with ITGB4 KD (MIA PaCa-2) exhibit reduced colony formation and are less sensitive to combination treatment with PH + 5FU + Oxali compared to single treatments (DMSO, PH, and 5FU + Oxali). Western blotting was performed to assess the expression of phosphorylated FAK (pFAK) and COL1A1 in PDAC cells with ITGB4 KD compared to non-silenced cells. **I** MIA PaCa-2 cells: ITGB4 KD results in a significant reduction in the expression of pFAK and COL1A1, suggesting that ITGB4 regulates key signaling pathways involved in cell adhesion and extracellular matrix remodeling. **J** KPC cells: Similar to MIA PaCa-2 cells, ITGB4 KD in KPC cells also leads to decreased expression of pFAK and COL1A1, further supporting the role of ITGB4 in regulating these critical pathways. Quantification of band intensity was performed and normalized to β-actin. Data are expressed as the mean ± SEM from three independent experiments. *p < 0.05, **p < 0.01, ***p < 0.001, and ****p < 0.0001 compared to control cells by two-way ANOVA and Two-tailed Student or Welch *t* test.

Next, we pursued to ascertain the mechanisms by which this combination treatment affects PDAC. We hypothesized that inhibition of ITGB4 expression contributed to the decrease in ECM markers overexpressed in PDAC. To further investigate this, we first performed immunofluorescence study and identified that, combination treatment significantly lowers the protein levels of ITGB4 and COL1A1 in both human (Fig. 5A, C, D) and murine PDAC cells (Fig. 5B, E, F) compared to DMSO, PH or 5FU + Oxali. We then analyzed the expression of ITGB4 in publicly available data sets and identified that, ITGB4 is highly expressed in PDAC (Fig. 6A) and its overexpression is associated with poor patient survival [23] (Fig. 6B). We then looked at our previous studies where we have performed the proteomics in the same murine model using gemcitabine instead of 5FU and Oxaliplatin [19]. Interestingly, we identified that, ITGB4 is significantly reduced in the combination of PH with gemcitabine compared to gemcitabine, PH or vehicle-treated alone (Fig. 6C). This suggests that PH is enhancing the efficacy of chemotherapy drugs by inhibiting the ECM formation through ITGB4. This hypothesis was supported by our findings that ITGB4 expression was significantly reduced in both gemcitabine and 5FU + Oxali treatments in combination with PH. We then validated the inhibitory effect of these combinations by performing western blot on tumor tissues (Fig. 6D) and cultured cells treated with the same conditions as in vivo experiment (Fig. 6E, F) and identified the similar reduction of ITGB4 and COL1A1.

To directly assess, if the ECM disruption in combination treatment was mediated by ITGB4, we genetically knockdown ITGB4 and observed the expression of COL1A1. Interestingly, the knockdown of ITGB4 showed the reduced growth in colony formation assay suggesting that ITGB4 is crucial for PDAC growth in both human (Fig. 6G and Supplementary Fig. 4A) and murine cells (Fig. 6H and Supplementary Fig. 4B). Furthermore, immunoblotting results in murine and human PDAC cells with ITGB4 knockdown showed reduced expression of phospho FAK (pFAK) and COL1A1, which is consistent with the combination therapy suggesting that genetic and pharmacological inhibition of ITGB4 inhibits the PDAC growth and ECM disruption through COL1A1 (Fig. 6I, J). These results collectively prove that the combination therapy disrupts expression of key elements of ECM such as COL1A1 by downregulating ITGB4, which in contributes to PDAC growth inhibition.

## Discussion

The addition of PH to 5FU + Oxali in PDAC significantly increased growth inhibition similar to the results previously reported with gemcitabine [19]. Increase in apoptosis as measured by annexin V and PI staining supports this is an underlying mechanism of inhibition [24, 25]. In orthotopic model, the combination exhibited significantly enhanced tumor growth inhibition compared to single treatments. The proteomic data supports increase in apoptosis pathways in the animal model like the observation in the cell lines. The unchanged bodyweight across all treatment groups indicates that this treatment does not have an increased toxicity profile, which is similar to the observed effects in gemcitabine-based clinical trials. Recent studies on clinical trials targeting ECM have not improved the drug delivery. For example, in a phase III trial evaluating PEGPH20, (hyaluronidase targeting hyaluronan), in addition to chemotherapy failed to meet its primary endpoint [26]. Our current data suggests that in the cases of tumors with dense stroma, such as PDAC, reprogramming the stromal compartments rather than depleting them through modulation of a single pathway may be a strategy that is more effective. Combination of chemotherapeutic agents with PH has the ability to modulate multiple stromal pathways and may represent a safer and more efficacious approach for evaluation in future clinical trials. The mechanisms underlying the enhanced anti-tumor effects in the animal model include modulation of ECM, changes in tumor immune microenvironment as well as alterations in DNA repair mechanisms.

PDAC microenvironment is immunosuppressive contributing to the aggressive behavior and resistance to therapy [27–29]. One of the most important aspects of our study is that the combination treatment alters the immune landscape in PDAC microenvironment. The activation of T NK cells and increased proportions of γδ T cells suggests that this combination not only targets tumor cells but also modifies the immune responses as well. The observed changes in the increased expression of CD3^+^ NK1.1^−^ PD-1^+^ and CD3^+^ NK1.1^−^ CTLA-4^+^ TNK cells indicate a favorable shift in the immune environment and favors the potential for combining this regimen with immune checkpoint inhibitors. The absence of significant changes in the CD4^+^ and CD3^+^ T cells could be because these populations are coupled with the activation specific regulatory cells (FOXP3^+^ PD-1^+^), suggesting a complex interplay where the combination treatment may promote regulatory pathways that could be combined for anti-tumor response.

ITGB4 is encoded with β4 subunit, which is a laminin receptor that exclusively interacts with α6 subunit and plays a vital role in the biology of infiltrating cancer [30]. ITGB4 overexpression plays a key role in cell adhesion by binding with ECM proteins to regulate the cellular functions by transmitting adhesion signals [31]. ECM is vital for developing resistant niches by permitting cancer cells to tolerate therapeutic drugs before mutagenic resistance mechanisms are acquired [32]. Overexpression of ITGB4 shows significant correlations with tumor development and metastasis in several cancers, including PDAC [33]. The molecular mechanism of this overexpressed ITGB4 in the ECM formation of PDAC is poorly understood.

Paricalcitol inhibits the expression of ITGA6, ITGB4, and α-SMA in human breast cancer cells [13]. H binds to collagens required for ECM formation [34]. H is a known autophagy inhibitor, and the autophagy is considered as important regulator of ECM deposition, fibrosis and tissue remodeling. Secretory autophagosmes also promotes ECM proteolysis [35]. The significant reduction in the ECM proteins, such as collagens, observed in our model supports our hypothesis that the combination treatment with PH disrupts the structural and biochemical ECM that supports that PDAC growth and metastasis. A candidate pathway that could underlie the observed ECM modulation is ITGB4. Increased expression of ITGB4 is correlated with EMT hallmarks, reduces the expression of E-cadherin, and increases the expression of vimentin [33, 36]. ITGB4 also known to be a key molecule in the interaction between PDAC and ECM and contributes to ECM formation. Supporting this role of ITGB4 in PDAC biology is the observed poor survival in patients with PDAC with increased expression of ITGB4. In this current study, the significant downregulation of ITGB4 by PH treatment suggested that the downregulation of ECM components, such as collagens or fibrinogen binding proteins, maybe driven in part by ITGB4. Genetic inhibition of ITGB4 demonstrated similar ECM disruption and inhibitory effects on PDAC growth. Given this novel finding, we evaluated effects of PH and gemcitabine from our prior study on ITGB4 expression. Similarly, a significantly reduction in ITGB4 was observed with the gemcitabine-based regimen, further confirming the role of this pathway in modulation of ECM observed after treatment with PH.

In conclusion, PH sensitizes PDAC to the effects of 5FU and oxali. PH has diverse effects on PDAC biology including immune modulation, autophagy, and inhibition of CAF. In addition, PH can modulate the ECM partly through ITGB4, which is a new mechanism of action of this regimen. The results observed in this study further support testing this regimen in future clinical trials.

## Supplementary information

Supplementary Figure legends

Supplementary Figures

SupplementaryTable 1

Supplementary methods

## Data Availability

All data and methodological details related to this manuscript are available upon request from the corresponding author. The corresponding author can be contacted for access to any supplementary information necessary for verifying or replicating the findings presented in this work.
