# Supplementary material for: Paricalcitol and hydroxychloroquine modulates extracellular matrix and enhance chemotherapy efficacy in pancreatic cancer
Source: Cancer Gene Ther. 2025 Sep 27;32(12):1330–40. doi: 10.1038/s41417-025-00967-9 (PMC12702779; doi:10.1038/s41417-025-00967-9)
Supplement: Supplementary file 1 — Supplementary Figure legends [file 41417_2025_967_MOESM1_ESM.docx]

**Supplementary Fig 1.** **PH potentiates the growth inhibition of 5FU, Oxaliplatin and in Irinotecan PDAC cells.**

**(A-C)** The indicated cancer cell lines were treated with various concentration of 5FU (15 μM) and Oxaliplatin (25 μM), Irinotecan (15 μM), paricalcitol (350 nM), and hydroxychloroquine (25 μM) for three days and subjected to MTT assays. Relative percentage cell viability was plotted with respect to DMSO treated cells. **(D)** The indicated cancer cell lines were treated with DMSO, PH, 5FU+Oxali+Irino, and combination (PH+5FU+Oxali+Irino) for 2–4 weeks, and long-term cell survival was measured using clonogenic assays. Representative images are shown. **(E)** Colony counts from the clonogenic assay data shown in 1D**.** Data represent the mean ± standard error of three biological replicates. ns = not significant, **p* < 0.05, ***p* < 0.01, ****p* < 0.001, and *****p* < 0.0001 compared to control cells by two-way ANOVA.

**Supplementary Fig 2.** Analysis of cell cycle progression by flow cytometry. Pictorial graph showing the proportion of cells in different phases of cell cycle treated with DMSO, PH, 5FU+Oxali and PH+5FU+Oxali treatments in MIA PaCa-2 (A), HPAC (B), and KPC (C) cells.

**Supplementary Fig 3.** **(A)** Proportions of CD4^+^ T-cells, **(B)** CD4^+^ Foxp3^-^ PD-1^+^ T cells, and **(C).** CD4^+^ Foxp3^-^ CTLA-4^+^ T cells in KPC TiME in DMSO, PH, 5FU+Oxali, and PH+5FU+Oxali treatments. **(D)** Expression of CD8+ T-cells, **(E)** CD8^+^ PD-1^+^ T cells, **(F)** CD8^+^ CTLA-4^+^ subsets from KPC TiME with the same treatment conditions as indicated above. Statistical significance was assessed using two-way ANOVA. ns = not significant, **p* < 0.05, ***p* < 0.01

**Supplementary Fig 4.** Quantification of clonogenic assay in NS and shITGB4 PDAC cells. **(A)** MIA PaCa-2 and **(B)** KPC cells treated with DMSO, PH, 5FU+Oxali, and PH+5FU+oxali. ns = not significant, **p* < 0.05, ***p* < 0.01, ****p* < 0.001, and *****p* < 0.0001 compared to control cells by two-way ANOVA.
