# Supplementary figures and images for: Paricalcitol and hydroxychloroquine modulates extracellular matrix and enhance chemotherapy efficacy in pancreatic cancer
Source: Cancer Gene Ther. 2025 Sep 27;32(12):1330–40. doi: 10.1038/s41417-025-00967-9 (PMC12702779; doi:10.1038/s41417-025-00967-9)

**Supplementary figures:**

**
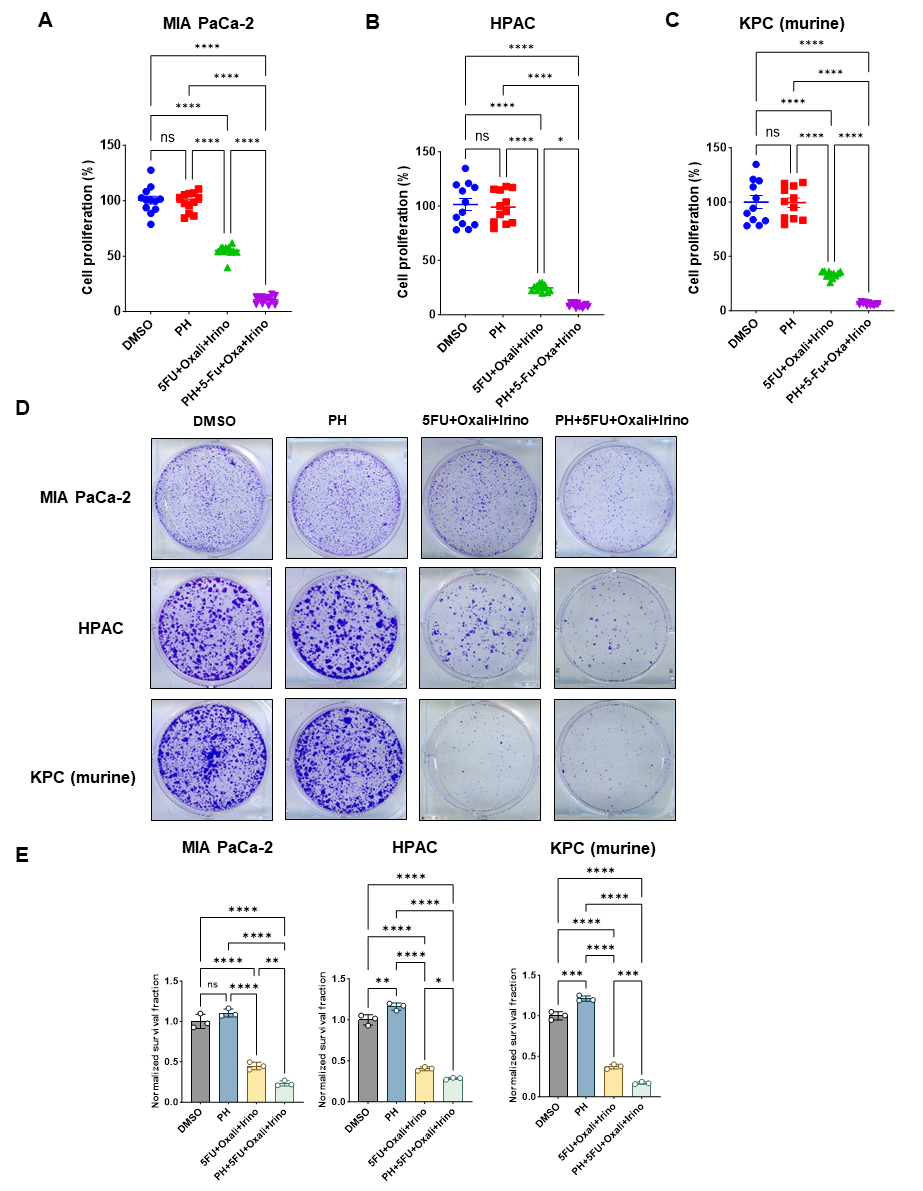
**

**
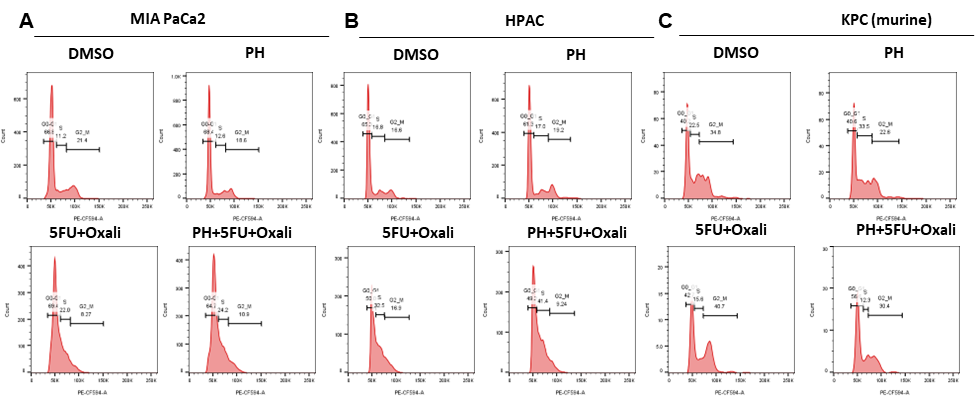
**

**
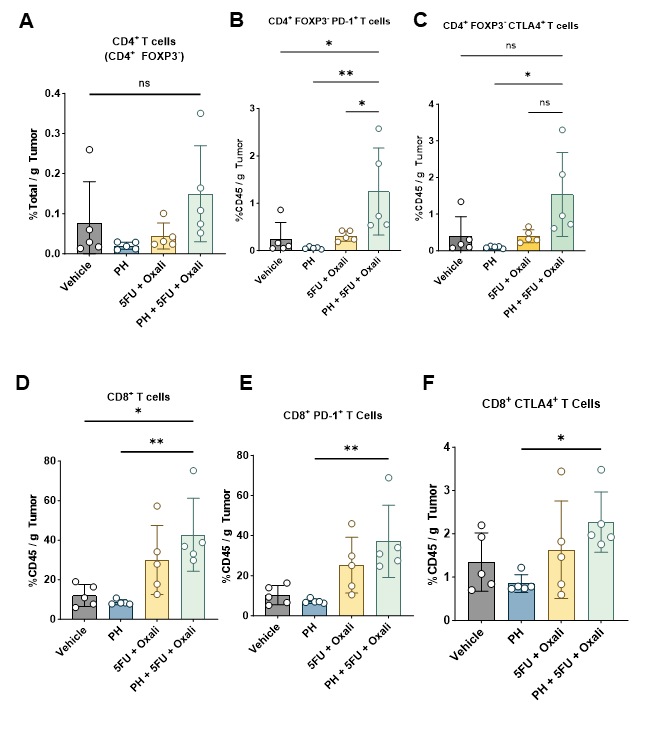
**

**
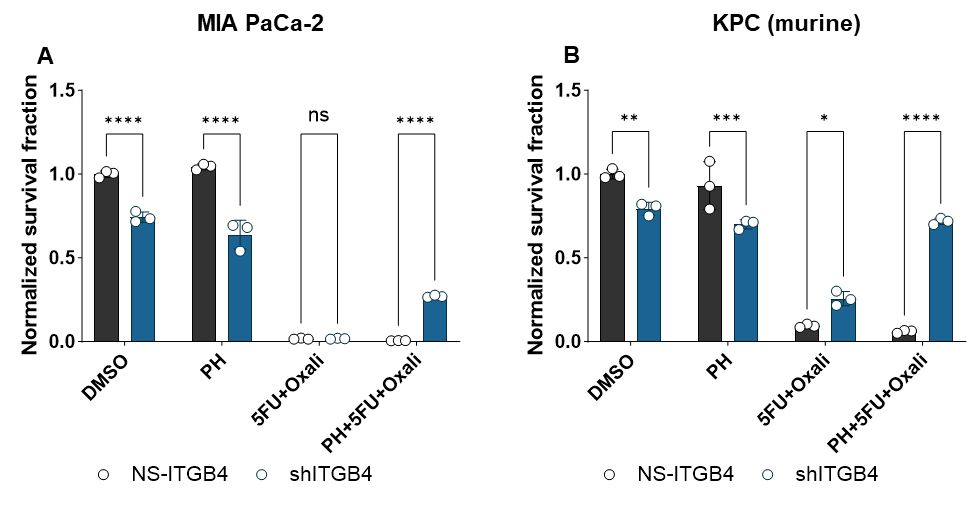
**

Supplement: Supplementary file 2 — Supplementary Figures [file 41417_2025_967_MOESM2_ESM.docx]
