# Supplementary material for: Paricalcitol and hydroxychloroquine modulates extracellular matrix and enhance chemotherapy efficacy in pancreatic cancer
Source: Cancer Gene Ther. 2025 Sep 27;32(12):1330–40. doi: 10.1038/s41417-025-00967-9 (PMC12702779; doi:10.1038/s41417-025-00967-9)
Supplement: Supplementary file 3 — SupplementaryTable 1 [file 41417_2025_967_MOESM3_ESM.docx]

| **Reagents** | **Source** | **Identifier** |
| --- | --- | --- |
| **Antibodies** | | |
| ACTINB | Santa-Cruz | sc-47724 |
| ITGB4 | Thermo Scientific | MA5-32608 |
| COL1A1 | Cell Signaling Technology | 72026 |
| pFAK | Cell Signaling Technology | 3283 |
| FAK | Cell Signaling Technology | 3285 |
| anti-mouse IgG, HRP-linked antibody | Cell Signaling Technology | 7074/7076 |
| **Chemicals, and Reagents** | | |
| DMEM | ATCC | 30-2002 |
| PBS | ATCC | 30-2200 |
| Fetal Bovine Serum | ATCC | 30-2020 |
| Horse Serum | Gibco | 16050-122 |
| Trypsin-EDTA | ATCC | 30-2101 |
| Penicillin-Streptomycin | Corning | 30-002 |
| DMSO | Sigma-Aldrich | D2438 |
| MTT | Millipore Sigma | 475989 |
| ProLong™ Gold Antifade Mountant with DNA Stain DAPI | Invitrogen | P36935 |
| RIPA buffer | Thermo Scientific | 89901 |
| BCA protein assay kit | Thermo Scientific | 23225 |
| 4-20% SDS gels | BIO-RAD | 4568096 |
| Bovin Serum Albumin | Fisher Scientific | BP1600 |
| Matrigel | BD bioscience | 354234 |
| PVDF membranes | Invitrogen | IB34001 |
| DNASE 1 | Fisher Scientific | NC9199796 |
| Collagenase IV | Fisher Scientific | NC9836075 |
| PE-Annexin V Apoptosis Detection Kit | BD Biosciences | 559763 |
| Propidium Iodide | Millipore Sigma | P4170 |
| **Experimental Models: Cell Lines** | | |
| KPC |  |  |
| Mia PaCa-2 | ATCC | CRL-1420 |
| HPAC | ATCC | CRL-1687 |
| **Experimental Models: Organisms/Strains** | | |
| Mouse C57BL6/J | The Jackson laboratory | 000664 |
| **Inhibitors/Drugs** |  |  |
| 5-fluorouracil | Selleckchem | S1209 |
| Oxaliplatin | Selleckchem | S1224 |
| Paricalcitol | Selleckchem | S6681 |
| Hydroxychloroquine | Selleckchem | E4824 |
| **Antibodies for Flowcytometry** | | |
| **Marker** | **Clone** | **Supplier-Cat #** |
| PD-1 FITC | 29F.1A12 | BioLegend, 135214 |
| CD45 PerCP | 30-F11 | BioLegend, 103130 |
| Foxp3 APC | FJK-16s | Invitrogen, 17-5773-82 |
| CTLA4 APC R700 | MP6-XT22 | BD, 565778 |
| CD3 BV 421 | 145-2C11 | BioLegend, 100341 |
| TCR γδ BV 605 | GL3 | BioLegend, 118219 |
| CD4 BV 650 | GK1.5 | BioLegend, 100469 |
| NK1.1 BV 711 | PK136 | BioLegend, 108475 |
| CD8 BV 785 | 53-6.7 | BioLegend, 100750 |

**Supplementary table 1.** Reagents and resources used in this study.
