# Supplementary material for: Paricalcitol and hydroxychloroquine modulates extracellular matrix and enhance chemotherapy efficacy in pancreatic cancer
Source: Cancer Gene Ther. 2025 Sep 27;32(12):1330–40. doi: 10.1038/s41417-025-00967-9 (PMC12702779; doi:10.1038/s41417-025-00967-9)
Supplement: Supplementary file 4 — Supplementary methods [file 41417_2025_967_MOESM4_ESM.docx]

**Materials and methods**

**Cell culture**

MIA PaCa-2 and HPAC cell lines were obtained from American Type Culture Collection (ATCC, Manassas, VA, USA) and maintained as recommended. KPC (LSL-Kras^G12D/+^, LSL-Trp53^R172H/+^, Pdx1-Cre) cell line was gifted by Dr. Gregory Lesinski (Emory University, USA) [1]. MIA PaCa-2 and KPC cells were grown in Dulbecco’s Modified Eagle Medium (DMEM; ATCC), supplemented with 10% fetal bovine serum (ATCC, Manassas, VA, USA) and 1% penicillin/streptomycin (ATCC) under 5% CO_2_. Additionally, MIA PaCa-2 cells received 2.5% horse serum. HPAC cells were grown in DMEM F-12 medium (ATCC) supplemented with 10% FBS and 1% penicillin/streptomycin in 5% CO_2_. All the cell lines were routinely tested for mycoplasma contamination to ensure the validity of the experimental results.

**MTT assay**

MIA PaCa-2, HPAC, and KPC cells were plated at a density of 5×10^3^ cells/well in 96-well plates. After 20 h, the cells were then treated with P (350 nM), HCQ (25 μM), 5FU (15 μM), Oxaliplatin (Oxali-25 μM), and Irinotecan (Irini-15 μM). After 72 h, the medium was removed, 10 μl methylthiazole tetrazolium (MTT; 5 mg/ml in PBS; Sigma-Aldrich, MO, USA) was added, and the cells were incubated for another 2 h at 37°C. The resulting formazan crystals were solubilized in 100 μl DMSO, and absorbance was measured at 570 nm with a reference wavelength of 630 nm.

**Clonogenic assay**

MIA PaCa-2, HPAC, and KPC cells were plated in 6-well culture plates (5 × 10^3^ cells/well). The cells were then treated with P (350 nM), HCQ (25 μM), 5FU (15 μM), Oxali (25 μM), and Irino (15 μM) for every 3 days. After 10 days, the cells were stained with a 0.005% Coomassie Brilliant Blue R-250 solution (Bio-Rad, USA), and the plates were imaged using an Epson Perfection V850 Pro Photo Scanner (USA). The resulting colonies were counted using image J.

**Apoptosis assay**

The binding of annexin V to cells was measured using the PE-Annexin V Apoptosis Detection Kit I (Cat #559763; Pharmingen, BD Biosciences, Franklin Lakes, NJ, USA), according to the manufacturer’s protocol. Briefly, KPC, MIA PaCa-2, and HPAC cells were treated with either DMSO, PH, 5FU+Oxali, or PH+5FU+Oxali for 72 h. After treatment, cells were collected, washed twice with cold 1X PBS, and resuspended in 1x Binding Buffer. The cells were then stained with 300 µL PE Annexin V FITC and 5 µL of PI and incubated for 15 min in the dark. Flow cytometry analysis was performed using a BD LSR Fortessa Flow Cytometer (BD Biosciences, San Jose, CA, USA) and data were analyzed using FlowJo software (Ashland, OR, USA).

**Cell cycle assay**

Following treatment with DMSO or PH, 5FU+Oxali, and PH+5FU+Oxali for 72 h, KPC, MIA PaCa-2, and HPAC cells were trypsinized and centrifuged at 1, 000 x g for 3 min at 4˚C. The cells were then washed with 1X PBS and fixed with 70% ethanol at 4 °C overnight. Following day, the cells were incubated with 1 ml of RNAse A solution for 30 min in dark and stained with propidium iodide for 30 min. following staining; cells were analyzed by flow cytometer (LSR fortessa, BD biosciences). The experiment was repeated thrice independently. The results were analyzed with FlowJo software (Ashland, OR, USA).

fection for 48 h, the U-2 OS and 143B cells

were harvested and xed in 70% ethanol at 4 °C overnight.

The next day, the cells were washed twice with PBS and

incubated with 0.2% Triton X-100 and 10 μg/mL RNase in

PBS at 37 °C for 30 min. Next, the cells were stained with

propidium iodide (PI, 20 μg/mL) in dark at 28 °C for 30

min. The stained cells were observed using a ow cyt-

ometer NovoCyte (Cat: 1300, ACEA, San Diego, CA,

USA). The experiments were performed thrice

independently

**Immunoblotting**

Whole-cell protein extracts were prepared using RIPA Lysis Buffer (Pierce Chemical, Rockford, IL, USA) containing Protease Inhibitor Cocktail (Roche, Basel, Switzerland) and Phosphatase Inhibitor Cocktail (Sigma-Aldrich, St. Louis, MO, USA). Lysed samples were centrifuged at 12,000 rpm for 40 min, and clarified supernatants were stored at −80 °C. Protein concentrations were determined using Pierce bicinchoninic acid (BCA) protein assay kit. Equal amounts of protein samples were electrophoresed on 4-20% sodium dodecyl sulfate (SDS)- polyacrylamide gels (BIO-RAD, #4568096) and transferred onto PVDF membranes (Invitrogen, #IB34001). The membranes were then incubated with antibodies diluted in 2% Bovine Serum Albumin (BSA, Fisher Scientific, #BP1600) for 2 h at room temperature. Primary antibodies were ITGB4 (Thermo Scientific #MA5-32608; 1:1000), phospho FAK (Tyr397) (Cell Signaling Technology #3283; 1:1000), total FAK (Cell Signaling Technology #3285; 1:1000), COL1A1 (Cell Signaling Technology #72026; 1:1000), and anti-beta actin (Santa Cruz #sc-47724; 1:3,000). Incubation with HRP-linked secondary antibodies (CST, #7074/7076;) at a dilution of 1:3000 in a 2% BSA solution was carried out for 1 h at room temperature. The signal was then detected on the LI-COR Odyssey DLx Imager using the ECL chemiluminescence detection system (Thermo Fisher Scientific, #34577).

**Immunofluorescence assay**

KPC and MIA PaCa-2 cells were grown on Falcon® 8-well culture slide (Corning, USA) at 40-50% confluence and treated with DMSO or PH, 5FU+Oxali, and PH+5FU+Oxali for 72 h. post treatment, the cells were fixed with 4% formaldehyde and permeabilized with PBS containing 0.2% Triton X-100 and 2% bovine serum albumin (BSA) for 30 min and then blocked with 2% BSA in PBS for 30 min. subsequently, the cells were then incubated with primary antibodies as follows: ITGB4 (1:100), and COL1A1 (1:100) for overnight. After being washed 3 times with PBS for 5 min each, the cells were incubated with appropriate second antibody (Alexa 568) for additional 1 h. Nuclear DNA was stained with Vectashield antifade mounting medium with 4,6-diamidino-2-phenylindole (DAPI) (VectorLaboratories). The images were obtained with Nikon A1R HD Confocal Microscope. The intensities were quantified using image J.

**Orthotopic grafting of PDAC cells in the mouse**

KPC-*f*-luc cells (1 × 10^5^) were suspended in Matrigel (1:1) in 35 μl of PBS solution and orthotopically implanted into the pancreatic tail of 4–5 week-old recipient C57BL6/J female mice. Tumor volume was measured by imaging every other week using the spectrum in vivo imaging system (PerkinElmer, Waltham, MA, USA). Total bioluminescence counts of the tumor-bearing areas were measured using the in vivo imaging software. The mice were randomized before the first imaging (i.e., pretreatment, Day 0) and were treated with vehicle (PBS) or PH (P 0.3 μg/kg; weekly twice) plus H (60 mg/kg; oral daily for fourteen days), 5FU+Oxali (5FU at 30 mg/kg plus Oxali at 5 mg/kg, weekly twice), and PH+5FU+Oxali. P, 5FU, and Oxali was administered intraperitoneally as mentioned above till the end of the experimental period. Body weights of the animals were measured once a week and at the endpoint, all the animals were subjected to imaging of whole body, euthanized and tumors were collected, weighed and used for subsequent experiments. The UAB Institutional Animal Care and Use Committee approved all protocols.

**Flow cytometry**

Tumor tissues from the previous experiments was minced and single cells were processed using gentleMACS dissociator (Miltenyl biotech). The cells were then washed with ACK lysis buffer to remove red blood cells. The cells were then filtered using 0.45 µm sterile filter and blocked with Fc blocking buffer. Followed by, the cells were stained with fluorophore conjugated primary antibodies (Table S1) and live/dead stain for 1 h in dark on ice. Then the cells were washed with PBS and suspended in eBioscience™ Foxp3 kit (catalog no. 00-5523-00) in the dark. Cells were then fixed using 2% Paraformaldehyde and filtered to remove debris before data acquisition. All the antibodies used in the present study were reported in Supplementary table 1. Flow cytometry (Symphony A5) was analyzed using the BD FACSymphony A5 cell Analyzer and FLOWJO software (version 10.7.2.).

**Proteomics sample preparation and result analysis**

Tissues samples were pulverized and were lysed in T-PER lysis buffer supplemented with protease inhibitors by dounce homogenization and incubation at room temperature for 15 min with gentle shaking. Following BCA protein assay, 40 ug each of samples were loaded onto 10% Bis-tris gels ½ way. The gels were stained overnight with Colloidal Coomassie. Each sample was digested with trypsin in 3 fractions overnight prior to LCMS analysis. Obtained proteins were queried by UniProtKB accession numbers to Reactome (V90) database pathways using the R package ReactomeContentService4R (v1.10.0) methods map2Events(), to obtain comprehensive lists of pathways, and get pathways(), to obtain the associated highest-level pathway, in R version 4.3.3. The second highest-level pathway categorization was obtained by recursing through the getEventsHierarchy() tree for Mus musculus [2]. Raw data from this study were submitted to the iPROX database and are available via ProteomeXchange with the identifier IPX0010315000.

**shRNAs transfection, lentivirus preparation, and stable cell line generation**

All shRNAs were obtained from SIGMA-Aldrich and are listed in the Supplementary table S1. Lentiviral particles carrying shRNA were generated by cotransfecting shRNA plasmids with the lentiviral packaging plasmids pSPAX2 and pMD2.G into 293T cells using Effectene (Qiagen) according to the manufacturer’s instructions. Culture medium was filtered using a 0.45-μm sterile filter to remove any dead or live cells and from lentiviral particles. Stable cell lines were generated by infecting MIA PaCa-2 and KPC cells with shRNA lentivirus in 12-well plates followed by puromycin selection (0.5–0.75 μg/ml for KPC and 2 μg/ml for MIA PaCa-2 cells).

**Statistical analysis**

GraphPad Prism (version 9.1.2, RRID: SCR_002798) was used for statistical analyses, and graphical representation with data are presented as either means ± SD or SEM. Two-tailed Student or Welch *t* test Two-way ANOVA with multiple corrections (Tukey or Sadik method) were performed to determine the statistical significance between groups. A *P* < 0.05 was considered statistically significant.

1. Mace TA, Shakya R, Pitarresi JR, Swanson B, McQuinn CW, Loftus S, et al. IL-6 and PD-L1 antibody blockade combination therapy reduces tumour progression in murine models of pancreatic cancer. Gut. 2018;67(2):320-32.

2. Milacic M, Beavers D, Conley P, Gong C, Gillespie M, Griss J, et al. The reactome pathway knowledgebase 2024. Nucleic acids research. 2024;52(D1):D672-D8.
